# Explorative Identification of Anatomical Parameters Associated with Successful Pessary Fitting in Pelvic Organ Prolapse Using Dynamic Magnetic Resonance Imaging

**DOI:** 10.3390/jcm13164819

**Published:** 2024-08-15

**Authors:** Charlotte P. R. Triepels, Lars L. Boogaard, Jurgen J. Fütterer, Sander M. J. van Kuijk, Wilbert A. Spaans, Roy F. P. M. Kruitwagen, Mirjam Weemhoff, Kim J. B. Notten

**Affiliations:** 1 Department of Obstetrics and Gynaecology, Maastricht University Medical Centre+, P.O. Box 5800, 6202 AZ Maastricht, The Netherlands; 2 3D Lab, Radboud University Medical Centre, P.O. Box 9101, 6500 HB Nijmegen, The Netherlands; 3 Department of Obstetrics and Gynaecology, Radboud University Medical Centre, P.O. Box 9101, 6500 HB Nijmegen, The Netherlands; 4 Department of Medical Imaging, Radboud University Medical Centre, P.O. Box 9101, 6500 HB Nijmegen, The Netherlands; 5 Department of Clinical Epidemiology and Medical Technology Assessment, Maastricht University Medical Centre+, P.O. Box 5800, 6202 AZ Maastricht, The Netherlands; 6 GROW—School for Oncology and Developmental Biology, Maastricht University Medical Centre+, P.O. Box 5800, 6202 AZ Maastricht, The Netherlands; 7 Department of Obstetrics and Gynaecology, Zuyderland Medical Centre, P.O. Box 5500, 6130 MB Heerlen, The Netherlands

**Keywords:** pelvic organ prolapse, pessary, dynamic magnetic resonance imaging, cystocele, explorative

## Abstract

**Background:** Pelvic organ prolapse (POP) affects many women and is often managed with pessary treatment, yet predicting the success of fitting remains challenging. This study aims to identify anatomical parameters associated with successful and unsuccessful pessary treatment using dynamic magnetic resonance imaging (dMRI). **Methods:** A cross-sectional study in Maastricht University Medical Centre (MUMC+), the Netherlands. Sixteen women with a cystocele and/or descensus uteri minimal POP-Q stage 2, using pessary treatment, were included. All women underwent a dynamic MRI of the pelvic floor at rest, during contraction and on Valsalva. The anatomical parameters evaluated included various lengths and angles. The association between the anatomical parameters and pessary fitted is assessed using partial least squares regression. The predictive accuracy was tested using cross-validation based on the partial least squares model with the most important variables. **Results:** Seven of the sixteen women (43.8%) were in the non-fitting group (due to movement, rotation or expulsion of the pessary), and nine women (56.3%) were in the fitting group. Participants in the non-fitting group had a significantly lower body mass index (BMI). Variables such as total vaginal length (TVL) and certain angles were highly predictive of pessary fitting success, with variable importance of projection (VIP) scores indicating their importance. The prediction models showed accuracies ranging from 53.3% to 80.0%. **Conclusions:** In this explorative study, TVL, cervical length (CL), sacrococcygeal angle and pubococcygeal angle were key variables associated with pessary fitting success. These findings offer valuable insights for optimizing pessary fitting procedures and the development of new pessaries.

## 1. Introduction

More than half of all ageing women develop pelvic organ prolapse (POP) of the bladder, uterus, rectum or a combination of these [1]. The reported prevalence of symptomatic prolapse is about 12% in women aged 45 years and above [2]. Treatment options for symptomatic POP include pessary treatment or surgery. Half of all women with symptomatic POP choose pessary treatment first [3]. Pessaries are available in different shapes and sizes. Shapes are often simple and symmetric and do not take into account the complex female pelvic anatomy. In 12–27% of women with POP, it is not possible to find a fitting pessary, and these women will undergo an operation instead [3,4,5,6,7,8]. A non-fitting pessary can be expelled through the introitus or is uncomfortable to wear, making pessary treatment unsuccessful. This is burdensome for the patient, ensures more consultations at the hospital and thus causes higher costs.

Previous studies showed different predictors of successful or unsuccessful pessary treatment [6,9,10,11,12,13,14,15,16,17,18]. The predictors for unsuccessful pessary treatment were as follows: a short vagina [6,7], a wide vaginal hiatus [7,13], genital hiatus to total vaginal length (GH/TVL) ratio > 0.8 [9], Valsalva genital hiatus area to ring pessary diameter (HARP) ratio > 5.00 [19], prolapse Pelvic Organ Prolapse Quantification (POP-Q) stage [9,10,13], levator ani avulsion [14], previous prolapse repair and hysterectomy [16,18], age 65 years or younger [9], older age [11], smoking [9], higher body mass index (BMI) [6,10,11], underactive pelvic floor muscles [11], predominant rectocele [17] and predominant anterior compartment prolapse [13,15]. 

Until now, it is unclear whether there are any anatomical parameters that may explain or predict why pessary treatment is successful or unsuccessful in women with POP. Understanding the relation between anatomical structures and pessary fitting in a dynamical situation is an essential step towards innovations in new pessary designs and treatment [20]. The aim of this exploratory study was to identify anatomical parameters of the pelvic floor in women with POP associated with a fitting and non-fitting pessary using dynamic magnetic resonance imaging (dMRI).

## 2. Materials and Methods

This cross-sectional exploratory study was performed in the Maastricht University Medical Centre (MUMC+), the Netherlands, and approved by the local medical ethics committee (METC152006). Symptomatic Caucasian women with a cystocele and/or descensus uteri minimal POP-Q stage 2 were recruited for participation. All women that were included had visited the clinic with the request for a pessary treatment or a clinical check of the pessary treatment. 

Women were excluded from participation if they had a history of prolapse or incontinence surgery, contra-indications for magnetic resonance imaging (MRI) or an isolated rectocele prolapse. Women with an isolated rectocele prolapse were excluded because it is known that, although a pessary remains in place, a rectocele often protrudes below the level of the pessary, and the pessary is not sufficient in treating symptoms of a rectocele [21].

Women in whom pessary treatment was successful for at least one year were grouped in the fitting group. A successful fitting was defined as the continued use without movement, rotation or expulsion of the pessary. Women for whom it was impossible to find a fitting pessary due to movement, rotation or expulsion of the pessary were grouped in the non-fitting group.

POP of minimal POP-Q stage 2 was defined as descent of the anterior vaginal wall, the posterior vaginal wall, the vaginal apex (uterine or vaginal vault prolapse) or a combination of these compartments, of which the most distal portion of the prolapse was ≤1 cm proximal to or distal to the plane of the hymen [22,23].

Three gynaecologists (KN, WS, GL) recruited the women during a follow-up consultation to evaluate the pessary treatment. The pessary was used for at least 6 months. All women gave written informed consent. The women filled in a questionnaire, underwent physical examination during a regular hospital visit and underwent a dMRI. Participants were examined at the first consultation by one of three experienced gynaecologists (KN, WS, GL) and staged according to the POP-Q [22,23].

Baseline characteristics included age, body mass index (BMI), parity, family history, history of hysterectomy, POP-Q stage, total vaginal length (TVL, assessed during physical examination), menopausal state and levator ani avulsion. A levator defect was classified as ‘major’ (more than half missing), ‘minor’ (less than half of the muscle missing) or as having no defect in the levator ani muscles, according to the classification system of DeLancey et al. [24]. 

All women underwent a dMRI without rectal or intravenous contrast. Participants were asked to empty their bladder prior to the examination. The first dMRI was performed without a pessary, followed by dMRIs with three different pessaries: ring, ring with support and Falk pessary. The correct size of the pessary was determined during a previous clinical visit at the hospital.

A gynaecologist gave the participant instructions during the dMRI to perform three different manoeuvres: a state of rest, contraction and Valsalva. A contraction was defined as one that resulted in bladder base elevation, and Valsalva resulted in bladder base depression on dMRI. The dMRI of the pelvic floor was performed in a 3T MRI scanner (MAGNETOM Skyra, Siemens Healthcare GmbH, Erlangen, Germany) with the participant in a supine position. A basic dMRI scan protocol for pelvic floor anatomy was used. T2-weighted images in the mid-sagittal and coronal planes were used. The duration of the dMRI was approximately 45 min.

**Figure 1:**
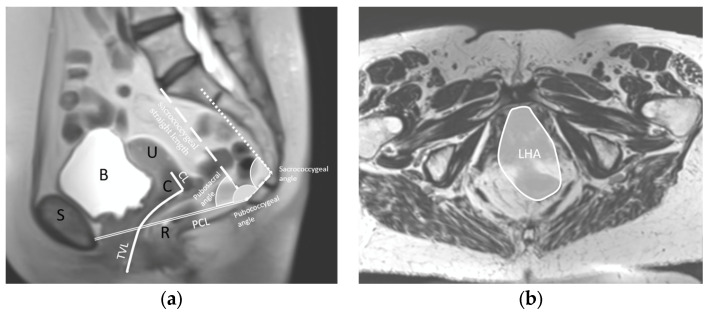
Definition of the anatomical parameters. (**a**) Sagittal view at rest with the total vaginal length (TVL), cervical length (CL), pubococcygeal line (PCL), pubococcygeal angle, sacrococcygeal angle, sacrococcygeal straight length and pubococcygeal–sacral angle. For reference, the pubic bone (S), bladder (B), uterus (U), cervix (C) and rectum (R) are denoted in black. (**b**) Coronal view at rest with the levator hiatal area (LHA).

The anatomical parameters were selected based on the literature [19,25,26] and on the clinical experiences of the researchers. The following parameters were assessed in the sagittal plane: pubococcygeus line (PCL) length, total vaginal length (TVL), cervical length (CL), pubococcygeal angle, sacrococcygeal angle, sacrococcygeal straight length and pubococcygeal–sacral angle (Figure 1). In the coronal plane, the levator hiatal area (LHA) was assessed (Figure 1b). Variables sacrococcygeal straight length and pubococcygeal-sacral angle were assessed at the three manoeuvres, only without a pessary. All other variables were assessed at the three manoeuvres, without the pessary and with the three pessaries. In total, 78 variables were assessed.

The records were assessed independently by two experienced examiners (JF) (CT). The examiners were blinded to the associated clinical data. In the case of there being a discrepancy between the measurements, they were discussed and measured a third time together. The third result was then used for analysis. All images were assessed offline using OsiriX Lite (version 10.0, Pixmeo, Geneva, Switzerland). 

Baseline characteristics are presented as mean and standard deviation (SD), median (range) or percentage. Differences between baseline characteristics between the two groups were assessed using the independent-samples *t*-test for continuous variables, Mann–Whitney U test for ordinal variables or Fisher’s exact test for nominal variables. *P*-values < 0.05 were considered statistically significant. Statistical analysis was performed using IBM SPSS for Windows/MAC, version 29.0 (IBM, Armonk, NY, USA).

Anatomical parameters associated with successful pessary fitting were assessed using partial least squares (PLSs) regression. PLSs regression is a statistical tool that constructs linear combinations of the original predictor variables while considering the response value.

All anatomical parameters were normalized using z-scores (centred to have a mean of 0 and scaled to have an SD of 1) and served as predictor inputs for the PLSs regression. The response input for the analysis was coded as −1 for a non-fitting pessary and 1 for a fitting pessary. Missing values were replaced with a value close to zero (1 × 10^−6^) after normalization to minimize their impact on the overall analysis while maintaining the integrity of the data distribution. 

In the initial analysis, we calculated the explained variance of the data as a function of the number of components included in the analysis and determined the variable importance in projection (VIP) scores. Variables with a VIP score greater than 1 were considered important in predicting the response [27].

Based on the initial analysis, we identified the most important variables using VIP scores. One to ten of the most important variables were then used as predictor inputs for a second PLS regression (validation study). To evaluate the prediction accuracy, we used cross-validation with one and two left out. The prediction response of the models was categorized as follows: if the prediction response was greater than 0.1, it was categorized as fitting. If the prediction response was lower than −0.1, it was categorized as non-fitting. Prediction responses between −0.1 and 0.1 were categorized as unknown to handle ambiguous cases. All calculations were performed using the statistical toolbox within MATLAB software (version R2020b; The Mathworks Inc., Natwick, MA, USA).

## 3. Results

Sixteen women with a cystocele and/or descensus uteri minimal POP-Q stage 2 were included. One woman experienced stress incontinence during pessary treatment and was initially placed in the non-fitting group. However, since the pessary was correctly fitted (remained in place during contraction and Valsalva) and because stress incontinence can be a masked condition that may also appear after pelvic surgery, we decided to include her in the fitting group for the analysis. Another woman could only complete the conventional static MRI due to claustrophobia. As a result, her data were incomplete and were excluded from the analysis. For the analysis, nine women were included in the fitting group and six in the non-fitting group.

The total study population had a mean age of 69.7 ± 8.5. years, and their BMI was 26.2 ± 3.9 kg/m^2^. Parity ranged from 1 to 3. Family history of POP was found in 40% (6/15) of the participants, hysterectomy was found in 13.3% (2/15) and 53.3% (8/15) had a POP stage of 3. The TVL was 8.4 ± 1.2 cm, 93.3% (14/15) were postmenopausal and 53.3% (8/15) had a levator avulsion (13.3% minor, 40.0% major), Table 1.

In the non-fitting group, there were more women with a POP-Q stage 3 compared to the women in the fitting group. In the fitting group, more women had a history of hysterectomy, and the women had a higher BMI, although this was not statistically significant. The two groups did not differ significantly in age, parity, family history of prolapse, total vaginal length (assessed during physical examination) and menopausal state. 

A total of twenty-eight variables had a VIP score greater than 1, five greater than 1.5 and one greater than 2. The VIP scores of the PLSs regression were the highest for the following variables: TVL during Valsalva without pessary (VIP: 2.33), TVL at rest without pessary (VIP: 1.88), CL at rest with ring with support (VIP: 1.75), sacrococcygeal angle during Valsalva with ring with support (VIP: 1.53) and TVL at rest with ring pessary (VIP: 1.50) (Table 2).

The accuracy (i.e., correct prediction) of the prediction models was found to be between 53.3% and 80.0%. The highest accuracy is found using six variables (80.0% (12/15) with one left out and 75.2% (158/210) with two left out. The prediction model, based on six variables, predicted incorrect in 20% (3/15) and unknown in 0% (0/15) with one left out, and incorrect in 21.0% (44/210) and unknown in 3.8% (8/210) with two left out. In both models, participants 7, 8 and 9 were mainly predicted incorrectly.

**Figure 2:**
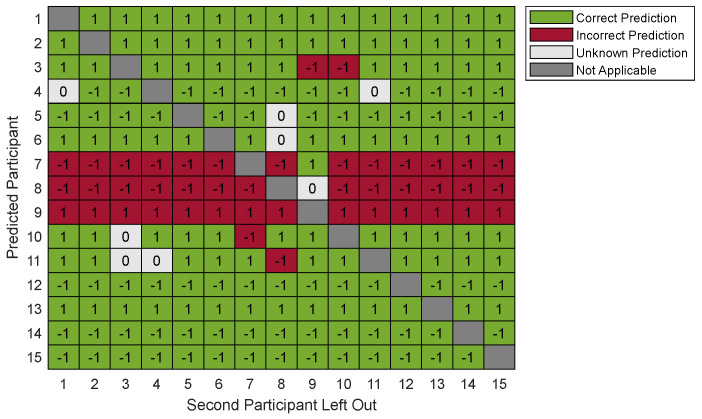
Heatmap of prediction accuracy of PLSs regression model with cross-validation (two left out, six variables). The boxes represent the prediction of each participant on the *y*-axis, while the corresponding participant was left out on the *x*-axis. The number inside each box represents the prediction, with 1 for fitting, −1 for non-fitting and 0 for unknown. A green box represents a correct prediction, red an incorrect prediction and white an unknown prediction.

Figure 2 provides a heatmap illustrating the prediction results using six variables and two left out, with a total of 210 combinations (14 combinations per participant). Participants 7 and 8 were predicted incorrectly in 92.9% (13/14) of the combinations, with two left out. Participant 9 was predicted incorrectly in all combinations (100%; 14/14). See Figure 2. 

## 4. Discussion

In this exploratory study, we found that the TVL (at rest and during Valsalva with or without a pessary), the CL (at rest with ring with support), the sacrococcygeal angle (during Valsalva with ring with support) and the pubococcygeal angle (at rest with ring) were the most important variables for predicting successful pessary fitting. Cross-validation yielded an accuracy of up to 80.0% in predicting correctly, with optimal use of six variables.

The mean vaginal length assessed during physical examination at rest was longer compared with the vaginal length assessed during dMRI (8.4 cm vs 7.3 cm, respectively). In the fitting group, a higher BMI was found compared to the non-fitting group. Therefore, we did not expect a higher BMI to increase the risk of unsuccessful pessary treatment.

A recent systematic review concluded that, among other factors, shorter TVL and larger HA on maximal Valsalva are associated with unsuccessful pessary fitting [26]. In our study, we found comparable TVLs assessed during physical examination between the fitting and non-fitting groups. Based on the PLSs regression, TVL (assessed on dMRI) is found to be an important parameter for predicting successful pessary fitting. The HA during Valsalva was not among the most important variables in our dataset. The VIP scores were 0.42, 0.92, 0.64 and 0.98 of HA during Valsalva without pessary, with ring, with Falk and with ring with support, respectively. Furthermore, several studies described levator damage as a risk factor for non-fitting pessary [13,28,29]. In our study, we found no statistical differences in levator damages between the groups.

To our knowledge, this study is the first attempt to investigate the association between anatomical parameters of the pelvic floor in women and the unsuccessful pessary treatment for POP using dMRI. Most studies focus on clinical and demographical parameters associated with unsuccessful pessary fitting. The dynamic evaluation with MRI of what happens and changes in situations of rest, contraction and Valsalva after the placement of a pessary reflects the changes that occur during the daily activities of a patient. 

In the United States, a Gellhorn pessary is often resorted to in instances where typical circular or disc-shaped pessaries are expelled. Frequently, these are effective because the stem keeps the disc portion from tipping. In the Netherlands, gynaecologists less frequently recommend the use of a Gellhorn pessary in the first attempt because of the higher risk of complications and limiting self-management. For this reason, this study only focused on ring, ring with support and Falk pessaries. 

The current study determined the most important parameters using PLSs regression. PLSs regression is particularly effective when the data have small samples or multicollinearity. Since we measured anatomical parameters during different manoeuvres and with different pessaries, they are very likely to be correlated. PLSs regression can manage this issue better than other regression methods [27]. The use of PLSs regression is well suited for exploratory studies and helps in identifying the most influential (important) variables without overfitting.

Generally, a variable with a VIP score greater than 1 is considered important. In our study, we found twenty-eight variables with a VIP score greater than 1, meaning the response (successful vs. unsuccessful) is explained by a large set of predictors. The high number of important variables indicates that the dataset contains a lot of variation and complex relationships.

The use of cross-validation for model validation ensures that the predictor variables are generalizable and provide a more reliable evaluation of its performance. Our prediction model, using six variables, mainly predicted participants 7, 8 and 9 wrong. Since those participants showed no outliers in the most important six variables, it is most likely that these participants contain different patterns compared to the other participants. These findings underscore the need for refinement of the model, possibly through larger studies. For the refinement of a prediction model, one should consider including variables derived from pre-treatment situations, as this could help identify baseline anatomical characteristics that contribute to successful pessary fitting.

Limitations of this study include the small size of the study sample. It is an exploratory study aiming to find clues for anatomical parameters that are worth studying more extensively as predictors for a fitting pessary. Secondly, in dynamic imaging on contraction and Valsalva, it is difficult to standardize the level of contraction and strength of a Valsalva. This can influence the measured increase in descent. The protocol was standardized, and the women were instructed and encouraged to give the maximal contraction and Valsalva they were able to. Lastly, it is worth mentioning that the anatomical parameters were assessed using dMRI, a technique that is not ideal in a clinical setting. It is not, however, our main goal to develop a clinically feasible prediction model. Our study aimed to provide insights into the anatomical factors that influence pessary fitting and could contribute to (1) improving our understanding of the working principle of pessaries. The underlying interaction between the identified anatomical parameters and pessaries is recommended for further study and (2) to boost the development of new pessary designs.

## 5. Conclusions

In this explorative study, key anatomical parameters influencing pessary fitting success were identified, with significant predictive accuracy achieved through PLSs regression models. Specifically, TVL, CL, sacrococcygeal angle and pubococcygeal angle were important parameters. The prediction models demonstrated accuracies between 53.3% and 80.0%, with the highest accuracy observed using six variables. These findings offer valuable insights for the development of new pessaries. In the case of developing and optimizing prediction models, the findings of this study should be considered, and the focus should lie on clinical applications.

## Figures and Tables

**Table 1:** Characteristics of the study sample. Independent-samples *t*-test of the means ± standard deviations, Mann–Whitney U test for median (range) and Fisher’s exact test for the percentages between the fitting group and non-fitting group. *n* = population size, BMI = body mass index, POP-Q = Pelvic Organ Prolapse Quantification, TVL = total vaginal length.

Baseline Characteristics		Total Study Population (*n* = 15)	Fitting (*n* = 9)	Non-Fitting (*n* = 6)	*p*-Value
Age (years) ^a^		69.7 ± 8.5	69.9 ± 9.8	69.3 ± 7.0	0.907
BMI (kg/m^2^) ^a^		26.2 ± 3.9	27.8 ± 3.8	23.9 ± 3.2	0.055
Parity ^b^		2 (1–3)	2 (1–3)	2 (1–2)	0.296
Family history of POP ^c^		40.0%	22.2%	66.7%	0.136
Hysterectomy ^c^		13.3%	11.1%	16.7%	1.000
POP-Q stage 3 ^c^		53.3%	33.3%	83.3%	0.119
TVL (cm) *^a^		8.4 ± 1.2	8.6 ± 1.5	8.2 ± 0.8	0.253
Postmenopausal (%)		93.3%	88.9%	100%	1.000
levator ani avulsion ^c^	No Avulsion	46.7%	44.4%	50.0%	1.000
	Minor	13.3%	11.1%	16.7%	
	Major	40.0%	44.4%	33.3%	

* Assessed during physical examination; ^a^ data expressed as mean ± standard deviations; ^b^ data expressed as median (range); ^c^ data expressed as percentage.

**Table 2:** The identified most important anatomical parameters based on the variable importance in projection (VIP) scores from the partial least squares (PLSs) regression, which were the input for the prediction model.

VIP Score	Anatomical Parameter	Manoeuvres	Pessary
2.33	Total vaginal length	Valsalva	None
1.88	Total vaginal length	Rest	None
1.75	Cervical length	Rest	Ring with support
1.53	sacrococcygeal angle	Valsalva	Ring with support
1.50	Total vaginal length	Rest	Ring
1.44	pubococcygeal angle	Rest	Ring

## Data Availability

The raw data supporting the conclusions of this article will be made available by the authors on request.
